# Fluid shear stress coupled with narrow constrictions induce cell type-dependent morphological and molecular changes in SK-BR-3 and MDA-MB-231 cells

**DOI:** 10.1038/s41598-020-63316-w

**Published:** 2020-04-14

**Authors:** Hamizah Ahmad Cognart, Jean-Louis Viovy, Catherine Villard

**Affiliations:** 1 Institut Curie and Institut Pierre Gilles de Gennes, CNRS, UMR168, Paris, France; 2 Université PSL, Paris, France

**Keywords:** Deformation dynamics, Single-cell imaging, DNA damage response, Lab-on-a-chip, Breast cancer

## Abstract

Cancer mortality mainly arises from metastases, due to cells that escape from a primary tumor, circulate in the blood as circulating tumor cells (CTCs), permeate across blood vessels and nest in distant organs. It is still unclear how CTCs overcome the harsh conditions of fluid shear stress and mechanical constraints within the microcirculation. Here, a minimal model of the blood microcirculation was established through the fabrication of microfluidic channels comprising constrictions. Metastatic breast cancer cells of epithelial-like and mesenchymal-like phenotypes were flowed into the microfluidic device. These cells were visualized during circulation and analyzed for their dynamical behavior, revealing long-lived plastic deformations and significant differences in biomechanics between cell types. γ-H2AX staining of cells retrieved post-circulation showed significant increase of DNA damage response in epithelial-like SK-BR-3 cells, while gene expression analysis of key regulators of epithelial-to-mesenchymal transition revealed significant changes upon circulation. This work thus documents first results of the changes at the cellular, subcellular and molecular scales induced by the two main mechanical stimuli arising from circulatory conditions, and suggest a significant role of this still elusive step of the metastatic cascade in cancer cells heterogeneity and aggressiveness.

## Introduction

Understanding the process of metastasis is a major challenge in the fight against cancer. This process is a multi-step one that often involves cells migrating from a primary tumor (at 90% of epithelial origin, i.e. carcinomas) into the blood stream (intravasation), where they reach a distant organ by re-crossing the endothelial barrier (extravasation). At both cellular and molecular levels, and in most cases, these events involve the ability of cells to undergo complex reprogramming processes. This involves in particular an epithelial-to-mesenchymal transition (EMT) at the primary tumor site, and a reverse process called mesenchymal-to-epithelial transition (MET), which may help in establishing distant micro-metastases after the circulation step^1–4^. Blood circulation, together with gene expression reprogramming, therefore plays a central role in the metastatic cascade.

Once in the bloodstream, circulating tumor cells (CTCs) experience shear stress. In addition, considering that capillaries do not have uniform diameters but are regularly constricted instead^5^, CTCs encounter various constraints within the microvascular network, leading to repeated mechanical deformation and eventually to arrest. Although numerous studies have revealed important influences of mechanical constraints in cancer^6^, surprisingly only a few works explored the responses of cancer cells to the mechanical stressors provided by the blood circulation, and most of them were limited to the study of the effect of shear stress. Malignant cells appeared more resistant than non-malignant cells to shear stress^7,8^ yet they still underwent significant apoptosis^9^, in particular as compared to hematopoietic cells^10^. Altered cytoskeleton organization in suspended and sheared primary ovarian cancer cells was observed^8^. Interestingly, the process of suspending cells in and of itself triggers myosin-II inhibition, leading to increased cell stiffness^11^. All these studies, however, involved flow-induced shear without topographical constraints, and relied on macroscopic tools that limited the control of the cell treatment uniformity, preventing single cell studies. The investigated biomarkers were also rather limited, targeting essentially cell survival.

Nonetheless, several works have used microfluidic systems to address the effect of the microvasculature geometry on cells. Pioneering work by Preira *et al*. investigated the role of cell deformability in the pathological arrest of leukocytes in the blood microcirculation of the lungs, by devising a microfluidic system with evenly spaced constrictions^12^. In the field of cancer, using circulated glioblastoma and normal glial cell lines, Khan *et al*. demonstrated the cell entry time into the confined space provided by 11 μm-wide diameter microchannels as a better marker of malignancy than deformability^13^. Au *et al*. showed that clusters of CTCs could reorganize reversibly in order to traverse microchannels of 5–10 μm-wide^14^. Nath *et al*. flowed HeLa cells across 7 μm-wide constrictions. They demonstrated that cell viability was reduced by 50%, but that the expression of MMP2, a metalloproteinase involved in stromal tissue degradation, was unchanged^15^. In a very interesting study, Xia *et al*. flowed leucocytes, MDA-MB-231 and MCF-7 cell lines into arrays of pores. They revealed a pressure dependence of the deformation of cells and nuclei, proposing that such studies could guide the optimization of CTC sorting devices^16^. Overall, microfluidic attempts at mimicking cancer cells in the blood circulation are still sparse, and are mostly focused on a phenomenological investigation of their mechanical properties. The question of the effect of blood circulation on the cell phenotype and its genome, and ultimately its aggressiveness is hardly addressed. The acquisition and maintenance of the mesenchymal phenotype, a key to the metastatic process require important cellular reprogramming by the activation of master regulators including transcription factors such as Snail, Twist and zinc-finger E-box-binding (ZEB), and transforming growth factor, TGF-β^17,18^. These phenotypic changes, however, are not bimodal, and recent studies suggest that disseminating tumor cells may present diverse and heterogeneous combinations of epithelial and mesenchymal phenotypic traits^14,19,20^.

The main aim of the present article is to tackle the least studied component of the metastatic cascade to date, which is the transient circulation step in the blood stream. More precisely, we explored the combined influence of shear stress and physical constraints on the characteristics of cancer cells put in motion by a flow recapitulating the pressure-velocity patterns of the blood microcirculation. We investigated, in particular, how circulation and constrictions regulate the phenotype, genome integrity and gene expression of these cells. Breast cancer cell lines with two different phenotypes, i.e. epithelial (SK-BR-3) and mesenchymal (MDA-MB-231), were circulated in microfluidic channels under pressure-regulated conditions. While under circulation, the tumor cells were constrained due to the presence of evenly-spaced multiple constrictions to mimic the mechanical condition in the blood capillary bed. The ways in which shear flow and mechanical constraints can promote changes in gene expression were investigated and elaborated based on the common framework for EMT and its transcription factors (EMT-TFs). Our results showed that circulation affects the cells at several scales, i.e. at cellular, sub-cellular and molecular levels, and that some of these changes are modulated by the epithelial or mesenchymal initial cell type. Therefore, the role of the circulation step seems to go beyond a simple disseminating function of cancer cells to distant organs. For this reason, it should be considered as an active step that is likely to modify gene expression of CTCs and possibly their mechanical properties.

## Results

### Biomechanical characterization of metastatic breast cancer cells under circulation, confinement and constrictions

**Figure 1:**
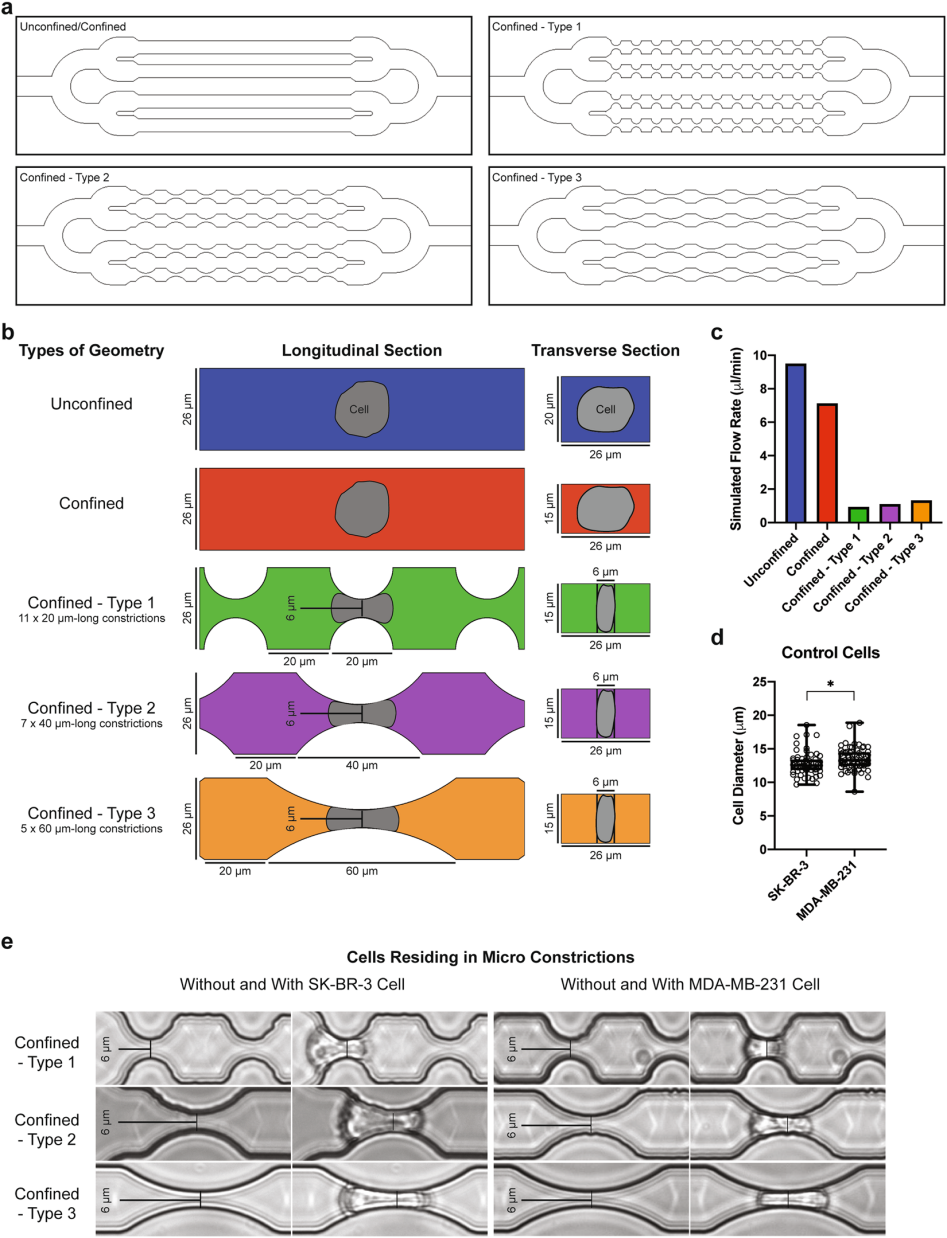
Types of geometry and hydrodynamics of the microfluidic system. **(a)** Schematic designs of microfluidic channels in full view. **(b)** Schematic designs of microfluidic channels with and without micro constrictions containing a representative cell body (grey shapes: the shape is only a guide for the eye). The longitudinal section is a cut by a plane parallel to the plane of the chip, encompassing the microchannel axis. The transverse section is a cut by a plane perpendicular to the axis of the microfluidic channel. The color codes for each types of channels will be used in data presentations for all subsequent figures. **(c)** COMSOL simulated flow rates of various microfluidic channels at a constant applied pressure of 10 kPa. **(d)** Cell diameter distribution of suspended control SK-BR-3 and MDA-MB-231 cells. Error bars represent standard deviation. Kolmogorov-Smirnov test. P value: 0.013. **(e)** Brightfield images of SK-BR-3 and MDA-MB-231 cells before and while residing in the three types of micro constrictions at a constant applied pressure of 10 kPa.

**Figure 2:**
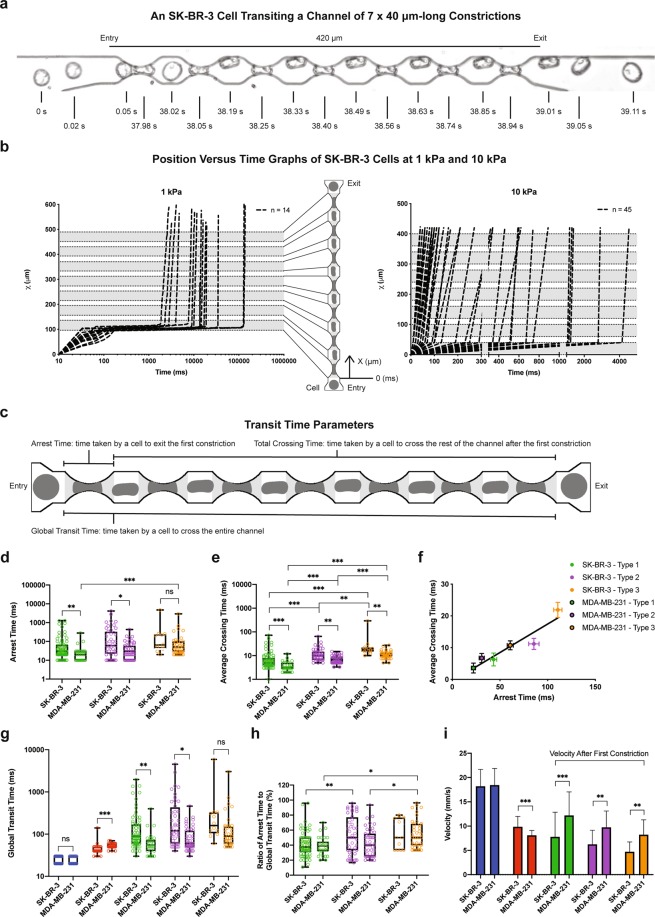
Transit of metastatic breast cancer cells in the microfluidic system (at a constant applied pressure of 10 kPa unless otherwise stated). **(a)** Overlay of a selection of images showing a SK-BR-3 cell transiting through a Confined – type 2 microfluidic channel at a constant applied pressure of 1 kPa with an acquisition rate of 100 frames per second. **(b)** Position versus time graphs of SK-BR-3 cells transiting through a Confined – type 2 microfluidic channel at 1 (left) and 10 (right) kPa. **(c)** Schematic trajectory of a typical cell (dark grey shapes) in a microfluidic channel using the Confined – type 2 geometry as an example in order to explain the different transit time parameters. The constrictions are indicated in light grey. **(d)** Box plot showing all points (minimum to maximum) of the arrest time of SK-BR-3 and MDA-MB-231 cells in the three types of micro constrictions. **(e)** Box plot showing all points (minimum to maximum) of the average crossing time after first constriction, defined as the total crossing time (see sub-figure (c)) divided by the remaining number of constrictions, of both cell types in the three types of micro constrictions. **(f)** Average crossing time as a function of the arrest time, for the three types of micro constrictions and for both cell types. **(g)** Box plot showing all points (minimum to maximum) of the global transit time of both cell types in various microfluidic channels. **(h)** Box plot showing all points (minimum to maximum) of the percentage of arrest time over global transit time of both cell types in the three types of micro constrictions. **(i)** Bar chart of the velocity of both cell types crossing the rest of the channel after the first constriction. Error bars represent standard deviation. For all data, the significance of results was analyzed using the Kolmogorov-Smirnov test. *significance level at 0.033; **significance level at 0.002; ***significance level <0.001; ns, not significant.

To test the effects of different geometric mechanical constrictions on the flow-induced migration of single tumor cells, five types of geometric microfluidic models were fabricated (Fig. 1). All five types comprised channels 420 μm in length (Fig. 1a). The “unconfined” type had a channel height of 20 μm, a value reduced to 15 µm in the “confined” types. Confined types – 1, 2 and 3 comprised channels with multiple (11, 7 and 5 respectively) 6 μm-wide constrictions spaced apart by chambers with a width of 26 µm and a length of 20 μm (Fig. 1b). Due to microfabrication constraints, the edges of the channels are not strictly perpendicular to the bottom surface of the device. The value of 6 µm is thus relative to the full width at half maximum of the height profile of our constrictions (Supplementary Fig. S1). These microfluidic designs were calculated for their flow resistance based on COMSOL simulations using the Stokes equation (the conditions correspond to a Newtonian fluid at low Reynolds numbers) (Fig. 1c, see also Supplementary Fig. S2). A pressure set-point of 10 kPa applied across the whole microfluidic circuit was chosen in order to achieve the same order of magnitude of flow rate (i.e. approximately 1 µl/min) as the typical blood flow rate reported in micron-sized capillaries *in vivo* (Supplementary Fig. S2)^21^. Either poorly (SK-BR-3) or highly (MDA-MB-231) metastatic breast cancer cell lines were delivered into these five types of geometric microfluidic models, for single cell mechanical phenotyping (Supplementary Fig. S3). These cells present approximately a diameter of 15 µm before circulation (suspended, uncirculated control condition, Fig. 1d), and are thus expected to undergo similar physical constraints in the circulation. For both cell types, constrictions trigger strong deformations, which increase with the constriction length, as expected from a crude volume conservation hypothesis (Fig. 1e). The cell path trajectories through the micro channels with constrictions were macroscopically scrutinized. Qualitatively similar behavioral patterns were observed for both cell lines and for two different pressure set points. A typical illustrative example is provided in Fig. 2a for SK-BR-3 cells in a type 2 constrictions array. A first quantitative observation is the large dispersion (i.e. over two orders of magnitude) of the “total transit time”, i.e. the total time spent in the constricted channels, whatever the applied pressure set point (Fig. 2b). Quite interestingly, the “position versus time” graphs of Fig. 2b reveal that the main factor limiting migration is the arrest in the first constriction. Once this constriction is passed, the subsequent ones are crossed smoothly with minimal arrest. The cell residence time in the first constriction (arrest time, see Fig. 2c) was then measured, as well as the crossing time (i.e. the time spent in the remaining part of the channel normalized by the number of encountered constrictions, see Fig. 2c) (Fig. 2d,e). The arrest and crossing times clearly highlight the differences associated with the three different constricted designs. The values of these two parameters increase significantly with the length of the constrictions (from 20 µm in the type 1 design to 60 µm for type 3). Comparison with our calculation of flow rates (Fig. 1c) shows that this cannot be explained by a difference in flow rates, since the latter increases from constrictions type 1 to constrictions type 3. Moreover, both arrest and crossing times are higher for SK-BR-3 as compared to MDA-MB-231 cells. Quite interestingly, the six conditions explored here (i.e. three types of constricted channels and two cells lines) fall on a master linear variation when plotting the crossing time as a function of the transit time (Fig. 2f). This suggests a common viscoelastic behavior for both cell lines, in which deformation and recovery are linearly related. As expected, the global transit time (see Fig. 2c), from entry to exit, display variations similar to those of the arrest and transit time, i.e. an increase with the constriction length and higher values for SK-BR-3 cells (Fig. 2g). The ratio between the arrest time and the global transit time (Fig. 2h) highlights the fact that cells spent 40% to 50% of their time in the first constriction, and this is even more pronounced when enlarging the length of the constriction. Lastly, cell velocities were evaluated (Fig. 2i). In the constricted channels, we took only into account the trajectory of the cells corresponding to the steep part of the curves displayed in Fig. 2b (i.e. after exiting the first constriction). First, all values are the range of a few mm/s in agreement with recent *in vivo* values reported in the literature^22^. Then, we observed lower velocities in the confined versus unconfined conditions, even corrected by the decrease of the flow rate between these two geometries (see Fig. 1c). This confirms that the interaction of cells with the top and bottom surfaces of 15 µm height channels (i.e. they are indeed confined) affect their velocity. Interestingly, this slow-down in the confined situation is more pronounced for MDA-MB-231 cells, a result consistent with the slightly higher diameter of these cells. Conversely, in constricted channels lower velocities were systematically measured for SK-BR-3 as compared to MDA-MB-231 cells. Finally, MDA-MB-231 cells flow at higher velocity in constricted channels as compared to non-constricted ones. This suggests that the bullet-like deformation that they undergo in the first constriction is an asset to easily cross the remaining part of the channel, including the subsequent constrictions. Overall, these consistent observations from Fig. 2d–i provide a picture, in which MDA-MB-231 cells are both more deformable and more plastic (in the sense that they keep a stronger memory of their deformation) than SK-BR-3 cells.

### Effects of circulation, confinement and constrictions on the morphology of metastatic breast cancer cells and their genome integrity

**Figure 3:**
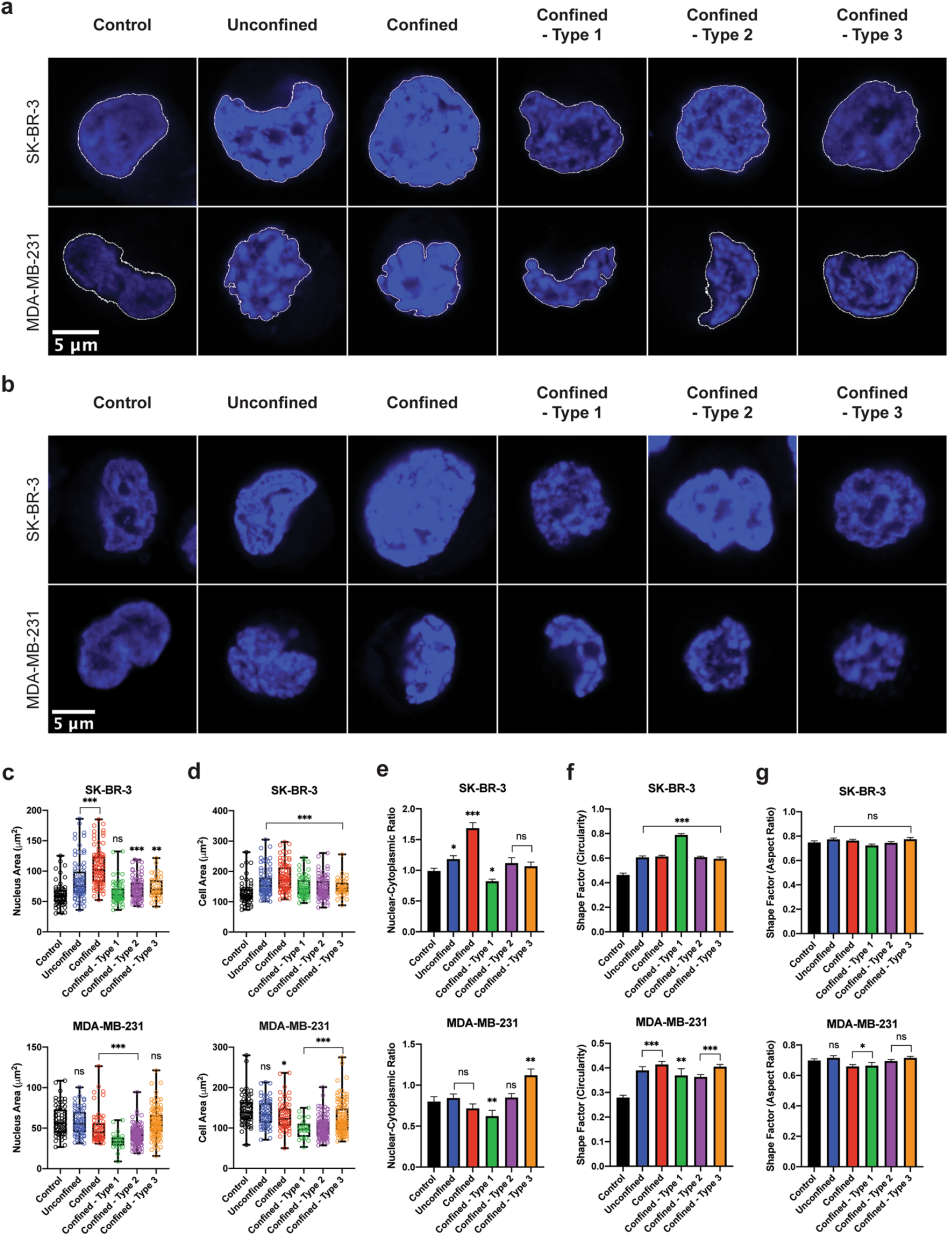
Morphological analysis of SK-BR-3 and MDA-MB-231 cells post-circulation, -confinement and –constrictions at a constant applied pressure of 10 kPa. **(a)** DAPI staining of the nucleus. Representative images of the mean values of nucleus area have been chosen for SK-BR-3 (top row) and MDA-MB-231 (bottom row) cells for each condition. **(b)** DAPI staining of the nucleus. Representative images of the mean values of circularity have been chosen for SK-BR-3 (top row) and MDA-MB-231 (bottom row) nuclei for each condition. **(c)** Box plot showing all points (minimum to maximum) of the nucleus area for both cell types. **(d)** Box plot showing all points (minimum to maximum) of the cell area for both cell types. **(e-g)** Bar charts of the N:C ratio, circularity of the nucleus and aspect ratio of the nucleus for both cell types respectively. Error bars represent standard error of mean. Kolmogorov-Smirnov test. *significance level at 0.033; **significance level at 0.002; ***significance level < 0.001; ns, not significant.

We then explored the consequences of the circulation on cellular morphologies. We focused most of our efforts on the nucleus, the biggest organelle in the cell, which was previously identified as the limiting one for cells in migration through narrow constrictions^23^. First, we characterized the nuclear morphological features of single tumor cells. Briefly, SK-BR-3 and MDA-MB-231 cells were isolated from the microfluidic system, fixed while in suspension, and their nuclei stained with DAPI (Fig. 3a,b, see also Supplementary Figs. S4, 5 for the images of representative subsets of nuclei). The maximum time over which cells are left in suspension, including the time spent before and after crossing the constrictions, is under an hour. Since each of the five geometric microfluidic models could impose unique mechanical constraint on the cells, their nucleus area, cell area and nuclear to cytoplasmic (N:C) ratio were quantified for each geometry (Fig. 3c-e respectively). In addition, the circularity (the ratio between the integrated area and the square of the perimeter) of the nucleus and its aspect ratio using Ferret minimum and maximum diameters were computed to get more insights into the cellular morphologies (Fig. 3f,g). Additional analyses, i.e. roundness and aspect ratio using an elliptic fit, are displayed in Supplementary Fig. S6. Morphological analysis showed significant increases of nucleus areas in SK-BR-3 cells in most conditions, with the highest changes observed as a result of circulation without confinement (channel height of 20 μm) and with confinement (channel height of 15 μm). This suggests that these cells respond strongly to being submitted to shear stress, and less to the additional strain provided by constrictions. This behavior is also confirmed by the variations of circularity and aspect ratio. Conversely, MDA-MB-231 cells appeared quite insensitive to the transition from a suspended to a circulated state, except regarding the circularity which increases significantly between both situations. They however, display significant changes in nucleus size when constricted. Let us finally note that type 1 constrictions seem to trigger more changes in morphologies as compared to other constricted conditions, in particular for MDA-MB-231 cells. It should be noted that, to keep the same total length of travel and the same length of chambers between constrictions, in this type the constrictions are shorter and sharper, but they are also more numerous (Fig. 1a). This repeated stress may amplify the consequence of mechanical deformation in plastic cells like MDA-MB-231 cells.

**Figure 4:**
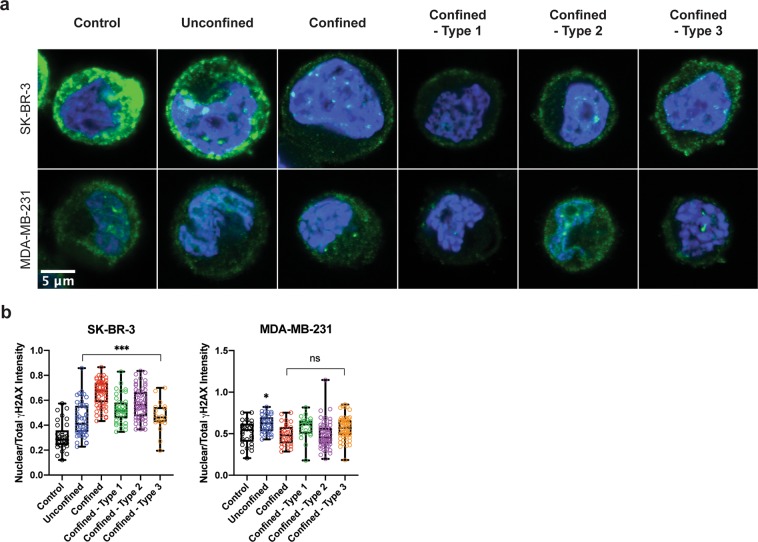
Genome integrity analysis of SK-BR-3 and MDA-MB-231 cells post-circulation, -confinement and -constrictions at a constant applied pressure of 10 kPa. **(a)** DAPI (blue) and γ-H2AX (green) staining of SK-BR-3 (top row) and MDA-MB-231 (bottom row) cells. **(b)** Box plot showing all points (minimum to maximum) of nuclear to total intensity of γ-H2AX for both cell types. Kolmogorov-Smirnov test. *significance level at 0.033; **significance level at 0.002; ***significance level < 0.001; ns, not significant.

Subsequently, to further investigate how the morphological changes observed in most circulated conditions as compared to the suspended control condition affect the genome integrity of the cells, the extents of DNA damage and repair activity due to circulation, confinement and constrictions were investigated by staining the cells with γ-H2AX (Phospho-Ser139) antibody, a marker for DNA damage response (Fig. 4a). The integrated densities of γ-H2AX in the nucleus and total cell fractions were measured and their ratio were represented (nuclear versus total γ-H2AX intensity, see Fig. 4b). These results showed that DNA damage and repair activity is significantly activated in SK-BR-3 cells in all circulated groups, as compared to the uncirculated (control) group. In MDA-MB-231 cells, DNA damage and repair activity is constitutively activated even in the control, and appears to be increased with a statistical significance (p = 0.045) only in the “unconfined” circulated case. Further analysis will be required to understand this point but we can note that a similar trend (also not statistically significant) is observed for the N:C ratio in size.

### Gene expression analysis of key regulators of epithelial-to-mesenchymal transition

**Figure 5:**
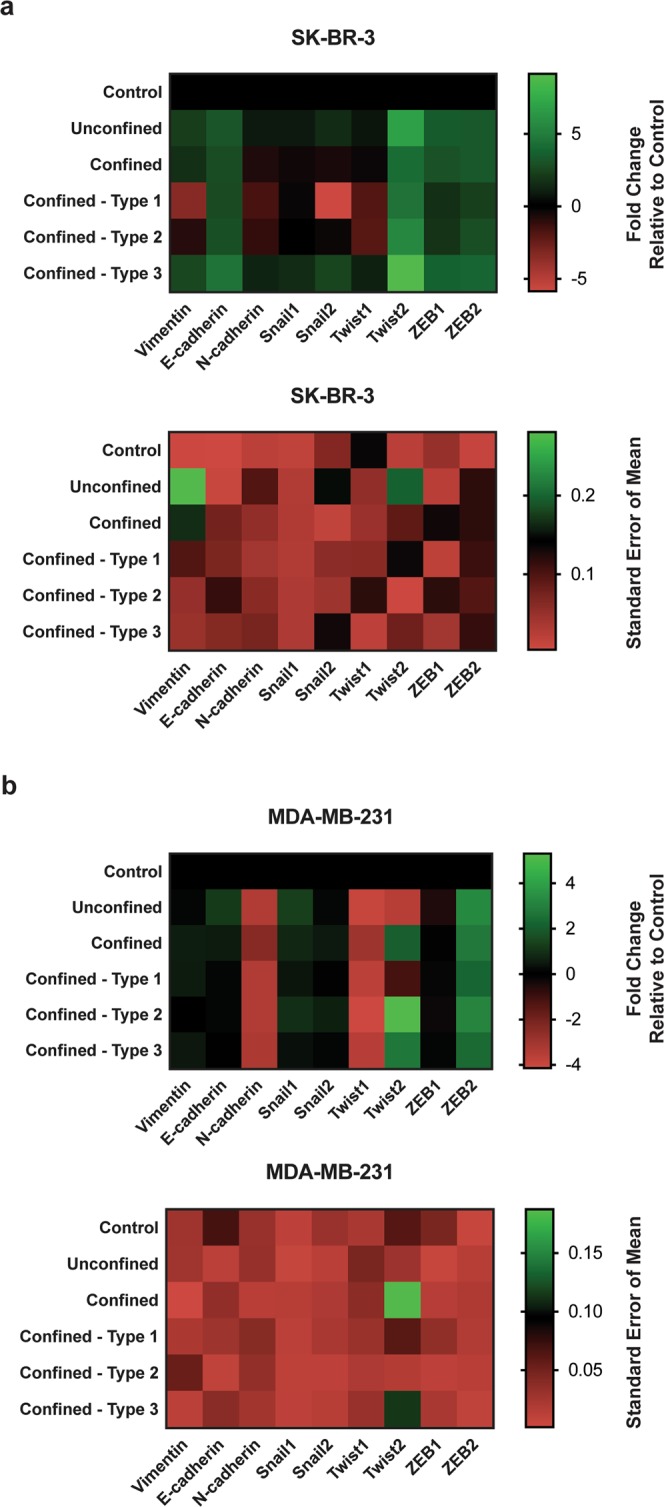
Heat map illustration of relative expression (log2, mean) and standard error of mean (normalized to the absolute mean) of Vimentin, E-cadherin, N-cadherin, Snail1, Snail2, Twist1, Twist2, ZEB1 and ZEB2 tested in two metastatic breast cancer cell types (n = 3 per transcript) in various microfluidic channels at a constant applied pressure of 10 kPa. **(a)** SK-BR-3 cells. **(b)** MDA-MB-231 cells.

Next, a panel of EMT transcriptional signatures was investigated for possible induced changes in tumor cells due to circulation, confinement and constrictions. Quite interestingly, significant changes in gene expression of EMT transcriptional signatures were observed in response to circulation alone (Table 1 and Fig. 5).

**Table 1:** Summary of statistical significance for the nine EMT transcriptional signatures tested in all conditions for both SK-BR-3 and MDA-MB-231 cells.

	SK-BR-3		MDA-MB-231	
	Non-circulated (control) to circulated (unconfined)	Circulated (unconfined) to constricted	Non-circulated (control) to circulated (unconfined)	Circulated (unconfined) to confined/constricted
Vimentin		− (**)		
E-cadherin	+ (**)			− (**)
N-cadherin			− (***)	
Snail1	+ (**)		+ (***)	
Snail2		− (***)		
Twist1			− (**)	
Twist2	+ (**)		− (*)	+ (***)
ZEB1	+ (***)			
ZEB2	+ (*)		+ (*)	

Kolmogorov-Smirnov test was used to compare relative expression of all transcripts in for cell type between two conditions/groups. “+” indicates up-regulation and “−” indicates down-regulation of relative expression of transcript. *Significance level at 0.033; **significance level at 0.002; ***significance level < 0.001; ns, not significant.

These changes are associated with an increase of expression in SK-BR-3 cells, 5 over 9 genes being significantly up-regulated (E-cadherin, Snail1, Twist2, ZEB1 and ZEB2). For MDA-MB-231 cells, a similar yet less pronounced trend was observed (i.e. only Snail1 and ZEB2 were significantly overexpressed), and notably, down-regulations of N-cadherin, Twist1 and Twist2 were observed (Table 1 and Fig. 5). When the effects of confinement and constrictions were added on top of circulation, no significant changes were detected for most of the transcriptional signatures investigated for both cell lines (Table 1). SK-BR-3 cells however, displayed significant down-regulated genes for Vimentin and Snail2 when compared to the unconfined circulated condition. Quite a different situation was observed for MDA-MB-231 cells in situations of confinement and strong deformations. Among the nine EMT transcriptional signatures tested, only E-cadherin was significantly down-regulated while Twist2, a transcription factor known to play a role in tumor progression in breast cancer^24^, revealed a striking increase in relative expression for all conditions (Fig. 5). Interestingly, Twist2 protein expression and nucleus to cytoplasmic localizations show also strong variations upon circulation in all confined states, with or without constrictions, as compared to the unconfined situation (see Supplementary Fig. S7).

## Discussion

Circulation of detached cancer cells within PDMS microchannels provides a foremost model to comprehend the fate of cancer cells during their transient existence in the blood circulation. In the present work, we have studied several aspects of this fate for two quite different cancer cell lines in terms of metastatic potential, SK-BR-3 and MDA-MB-231.

In suspended, control conditions, MDA-MB-231 cells display slightly more irregular (i.e. lower mean circularity value, p < 0.001) nuclei than SK-BR-3 cells for similar nucleus areas (p = 0.616). Then, the nuclear morphological analysis revealed striking differences between these cell types post-circulation in which nucleus area and circularity emerge as very responsive parameters. Globally, both cell types responded inversely to confinement and to the strong deformation induced by constrictions regarding the projected cellular areas: SK-BR-3 cells display marked increases in nucleus and cell areas, while MDA-MB-231 cells show a significantly opposite behavior, which might be reminiscent of the auxetic response described in the case of stem cells, i.e. of a cross-sectional contraction following compression leading to a reduction of the nucleus size^25^. This global trend in cellular morphology goes along with different dynamics of these cells after passing through the first constriction: SK-BR-3 cells tend to flow slower than MDA-MB-231 cells. Besides, due to their similar nucleus size in control condition, one might have expected that both cell types have similar responses in terms of arrest time in the first constriction. Interestingly however, values of arrest times are significantly higher in SK-BR-3 cells. They are consistent with the “transportability coefficients” computed by Liu *et al*., showing that MDA-MB-231 cells are much more deformable than SK-BR-3 cells^26^. Our results are therefore in line with the accumulation in the literature of evidence showing that the deformability of both cells^27^ and nuclei increases with their metastatic potential^28^.

Overall, our data regarding both cell dynamics at very short scale and final cell morphology after passing through the constrictions raises the issue of the link between nuclear stiffness and plasticity. Recent experimental^29^ and modeling^30^ studies have shown that softer nuclei (i.e. Lamin A/C-deficient nuclei in the work of Cao *et al*.) present a larger irreversible plastic deformation after being constricted than stiffer nuclei. However, it is not straightforward to examine the situation of fast cell deformation under flow in the light of these previous results, since the latter were established for the spontaneous migration of adherent cells. We hope that experimental studies, including the present work, might stimulate dedicated modeling approaches. It would be for instance useful, for e.g. the observation that a significant nucleus area decrease is observed for type 1 design compared to either unconfined or confined conditions, to have a conceptual framework to understand how the geometry of applied strain may have an impact on cell rheology at long time scales (i.e. minutes and above). We can already state, however, that our data highlight differential mechanical characteristics under shear flow and pressure-driven deformation, qualitatively similar to previous observations in spontaneous migration, of highly metastatic cancer cell lines being more deformable and plastic than less metastatic ones.

In the literature, the mechanical phenotype of cells is emerging as a potential biomarker for cell type classification, particularly cancer cells. Many studies have reported the use of microfluidic devices to measure the mechanical phenotypes of cells, including transit time, entry time, cell size, elastic modulus and cell fluidity^12,31–33^. This was applied to dissociated cells from tumors, in line with previously proposed deformation assays^34,35^. We believe that this is particularly interesting for CTCs, since they are expected to undergo large mechanical constraints during their crossing of microcapillaries *in vivo*.

Studies on DNA damage response (DDR) to mechanical stress, particularly fluid shear stress, mechanical confinement and constrictions are still scarce^36^, yet they are slowly emerging within the last years. DDR is a series of coordinated responses designated to remove damage incurred to the genome^37^. Using transformed cell lines, Singh *et al*. reported that lamins A/C, essential nuclear envelope proteins, are required for maintaining genomic stability and that their depletion stalls DDR^38^. Later, Davidson *et al*. associated the role of lamins A/C and DNA damage with migration-induced nuclear deformations in fibroblasts^23^. Several additional studies emerged, reporting that the genomic instability caused by migration-induced nuclear deformation and DNA damage promotes heterogeneity of the cancer cell genome^39–41^. This establishes an interesting interplay between nuclear mechanics, genome integrity and phenotypic transformation. In the present study, tumor cells that underwent circulation, confinement and constrictions indeed were found to present increased levels of DNA damage marker, γ-H2AX (Fig. 4). As compared to MDA-MB-231 cells, SK-BR-3 cells exhibited more significant increases in nuclear localization of γ-H2AX. Simplistically, this could suggest that DDR is more weakly activated by physical mechanical constraints in MDA-MB-231 than in SK-BR-3 cells. Upon closer inspection, however, DDR is almost twice higher in MDA-MB-231 (mean ratio of nuclear versus total γ-H2AX intensity: 0.5130) than in SK-BR-3 (mean ratio of nuclear versus total γ-H2AX intensity: 0.3088) cells at baseline, i.e. prior to flow. COSMIC, the Catalogue of Somatic Mutations in Cancer (http://cancer.sanger.ac.uk) reported 4 gene mutations in SK-BR-3 cells and a whopping 174 gene mutations in MDA-MB-231 cells^42^. This may imply that DDR mechanisms are more active in MDA-MB-231 than in SK-BR-3 cells due to a higher number of gene mutations, already in the absence of stress. The specific contribution of cell deformation to DDR thus may appear smaller in relative value in MDA-MD-231, simply because other non-mechanical causes of activation are already more active. Our results, however, may also reflect a more fundamental property of mesenchymal-like cells, i.e. a better ability to deform without inducing further DNA damage.

The plasticity of EMT in cells from aggressive tumors enables them to switch from proliferative to invasive phenotypes and vice versa^43,44^. It has been vastly observed in mouse mammary tumor models that the initiation of EMT can occur during the early stages of tumorigenesis and progress during its later stages^45–48^. The EMT process is considered as a hallmark-facilitating program as well as having influence on pathways linked to tumor progression and metastatic dissemination. Recent studies indicate that EMT occurs in a gradual manner characterized by several cellular states, exhibiting intermediate morphological, transcriptional, and epigenetic features^49^. These intermediate states between epithelial and fully mesenchymal states have been referred to as hybrid EMT states (see e.g. for a review)^50^. CTCs with hybrid epithelial/mesenchymal phenotypes were found in the blood of cancer patients and were associated to poor clinical prognosis^51^. Interestingly, it has been reported that hybrid EMT profiles increase cell survival in suspension^52^.

Our first general observation is that the changes in gene expression induced by the circulation itself are more pronounced than that of confinement and constrictions imposed upon already circulating cells. This result echoes and consolidates a recent study showing that the suspension state of breast cancer cells, including MDA-MB-231 and SK-BR-3 cells, promotes their metastatic potential^53^. Constrictions, however, also have specific effects at the expression level: four genes showed significant variations when the cells are submitted to confinement or constrictions (i.e. Vimentin and Snail2 for SK-BR-3 cells, and E-cadherin and Twist2 for MDA-MB-231 cells). Overall, we found that both epithelial and mesenchymal markers can be over-expressed in the same group of cells, and for both cell types. This joined up-regulation of epithelial and mesenchymal markers is in line with evidences of the existence of hybrid mesenchymal-epithelial states, suggesting that the circulation step might participate to the expression of this complex intermediate EMT.

In this study, it was observed that epithelial-like SK-BR-3 cells underwent different changes in morphological features, DDR mechanisms and gene expression as compared to mesenchymal-like MDA-MB-231 cells after exposure to mechanical stress from fluid shear stress and mechanical constraints. These findings suggest that circulation may have a selective effect on a population of CTCs with a pre-existing heterogeneity, in terms of survival (more detrimental to epithelial-like phenotype). It may also affect their metastatic potential at the circulation level, regarding e.g. the penetration in capillaries of organs (favoring the more flexible mesenchymal phenotype), and/or cell arrests leading to extravasation (oppositely favoring the more rigid epithelial one). Then, both epithelial and mesenchymal-like cells undergo in the crossing of micro-constrictions long-lived mechanical deformations. This remanence of the deformations may certainly help these cells to invade the microcapillaries of distant organs, but they may also play a role in the extravasation process, and in the migration of the extravasated cells in the invaded tissues. More generally, the demonstration of significant changes in the phenotype of cells during their circulation, indicates that the circulation step can in itself, and beyond the direct effects on extravasation discussed above, modify their behavior and aggressiveness in the invaded tissues. Without any doubts, our *in vitro* system is a very minimal mimic of blood micro-vessels, and its realism could be further improved. As it is, however, it recapitulates the “first level” basic mechanical constraints provided by the microvasculature: shear stress through flow, and repetitive constrictions. This work unambiguously shows cellular morphological and molecular responses to these cues.

In summary, flowing cancer cells from cell lines in micro channels, in conditions tailored to mimic those encountered in the blood flow, has shown significant impacts on cellular processes that can lead to changes in morphological features and genetic expressions. These changes certainly play a role in the migration of these cells, but they may also affect their fate and aggressiveness during extravasation and beyond, a possibility that will deserve further investigation. Also, two cell lines presenting an epithelial-like and mesenchymal-like state, respectively, have different responses. Therefore, this work also suggests that the effect of mechanical process of circulation in the blood flow is cell type-dependent. Since circulation in the blood is a necessary step in a large fraction of metastatic dissemination events (except for dissemination through the lymphatic system), these findings suggest that this step should not be ignored when trying to comprehend the metastatic process as a whole. This should help to achieve a more in-depth understanding of the molecular mechanisms activated or repressed in CTCs in the blood microcirculation from hydrodynamic shear stress and mechanical deformations, and how these circulation-induced changes can tune the metastatic potential of CTCs. In particular, it should be an important step to consider, in the investigation of cancer cell population heterogeneity, and on the consequences of this heterogeneity for treatment and patient’s fate.

## Methods

### Photo-masks design

A computer-aided design software application for two-dimensional design and drafting called QCAD was used to design different types of geometry (Fig. 1a). The total length of one geometrical channel is 420 μm. Each type of design consists of four 420 μm-long channels that are arranged in parallel. A control design without constrictions has a channel width of 26 μm. All constrictions have a width of 6 μm at its narrowest. Three types of designs with different constrictions lengths were prepared. The first type contains 11 constrictions with each measuring 20 μm in length (confined – type 1). The second type contains 7 constrictions with each measuring 40 μm in length (confined – type 2). The third type contains 5 constrictions with each measuring 60 μm in length (confined – type 3). The constrictions are connected with a transit channel 20 μm by 26 μm in dimension (Fig. 1b).

### COMSOL simulation

Before materializing the microfluidic designs, they were calculated for their flow resistance using COMSOL Multiphysics Modeling Software. The Stokes equation, a condition that corresponds to a Newtonian fluid at low Reynolds numbers, was applied in order to simulate the flow rates of these microfluidic designs at a constant applied pressure of 10 kPa (Fig. 1c, see also Supplementary Fig. S2).

### Optical photolithography

The above two-dimensional geometrical designs were etched onto a glass substrate with a chrome layer. The production of this chrome mask was outsourced from Selba S.A., Switzerland. From this chrome mask, a silicon master mold was fabricated in a cleanroom where provisions were made to reduce particulate contamination and control other environmental parameters such as temperature, humidity and pressure. A silicon wafer was used as a substrate. A negative photoresist SU-8 is spin-coated onto the substrate. SU-8 2010 photoresist was used to obtain structures with structure heights of 20 μm and 15 μm. After spin-coating the substrate with SU-8 2010 photoresist, the photoresist-coated substrate is soft baked at 95 °C (Supplementary Table S8a). An optical lithography mask aligner called MJB4 (SÜSS MicroTec AG, Germany) was used to expose UV light unto the chrome mask that was aligned on the photoresist-coated substrate. The UV light exposure dosage was optimized for each of the desired thickness (Supplementary Table S8b). Post exposure bake at 95 °C is carried out directly after exposure. The exposed photoresist was then developed in a shaking bath of photoresist developer (Supplementary Table S8c). After development, the developed image was rinsed with fresh developer solution for approximately 10 seconds, followed by a rinse with Isopropyl Alcohol (IPA) for another 10 seconds. Then, it was air-dried with pressurized air and hard baked for 30 minutes at 300 °C. The fabrication of a silicon master mold is now complete. The fabricated constrictions were measured for their precision using an Optical Profiler. Supplementary Fig. S1 shows a cross-section of the constriction (narrowest part of the channel), and that the value of 6 µm is achieved relative to the full width at half maximum of the height profile of the constriction.

### Soft lithography

From this silicon master mold, a microfluidic polydimethylsiloxane (PDMS) chip is fabricated through soft lithography. PDMS silicon elastomer, Sylgard 184 was purchased from Dow Corning (Michigan, USA). It was mixed with the provided curing agent at a ratio of 9:1 and then poured over the silicon master mold. They were cured at 70 °C and left to harden for 4 hours. After curing, the PDMS stamp was separated from the silicon master mold. 1.5 mm holes were punctured on the designated areas of the PDMS stamp in order to create reservoirs. Oxygen plasma treatment was performed to induce permanent bonding and the chip was closed by a glass coverslip. From this, PDMS-bonded slides were used as chips for the microfluidic system.

### Microfluidic system and image acquisition

All reagents and cell suspensions used during the experiments (Supplementary Fig. S3) were stored in pressurized containers that were connected to the microfluidic chip through a manifold valve (Fluigent, France). An MFCS-8C Flow Controller (Fluigent, France) was used to control the pressure independently in each container to drive all reagents and cell suspensions into the microfluidic chip. A heat generator was used to warm the microfluidic device at 37 °C throughout the experimental duration in order to achieve optimal culture conditions for experiments with live cells. Each microfluidic chip experiment experienced three major stages. The first stage is the priming of the chip in which the microfluidic channels were sterilized with 70% Ethanol (EtOH) followed by a washing step with 1X Phosphate-Buffered Saline (PBS). In order to have non-adhesive surfaces, the channels were coated with 100% of 1 mg of PLL-g-PEG (Susos AG, Switzerland) solution that was dissolved in 100 mM of sodium bicarbonate (NaHCO3) buffer at pH 8.5 for 1 hour.1 The channels were washed with 1X PBS and then filled with serum-free Dulbecco’s modified Eagle’s medium (DMEM) with HEPES (ThermoFisher Scientific, USA). The intermediate stage is the introduction of cells into the chip. A suspension volume of approximately 5000 cells in serum-free DMEM with HEPES was introduced into the microfluidic chip. This approximation of cell number was deemed optimal for the microfluidic models used in order to optimally observe single cell flow-induced migration in the channels without cell clumping or channel clogging. Here, single cell flow-induced migration was recorded based on the image acquisition control of frame rate. The image acquisitions of cells crossing the micro channels of constrictions at 100 frames per second were produced using the Basler acA800–510um USB 3.0 camera (Basler AG, Germany). The final stage is the retrieval of cells from the chip. Following the introduction of cells into the microfluidic chip, circulation of cells was allowed for 1 hour. Then, the cells were carefully retrieved from the output reservoir for two follow-up tests. The final volume of retrieved cells from one microfluidic experiment was approximately 10 μL. The first follow-up test was for molecular genetic analysis and the second follow-up test was for immunofluorescence (IF) analysis of cellular proteins.

### Cell tissue culture

All cell lines were purchased from American Type Culture Collection (Virginia, USA). All cell culture reagents were purchased from Gibco, ThermoFisher Scientific (Massachusetts, USA). Human breast cancer cell lines: SK-BR-3 and MDA-MB-231 were cultured in complete Dulbecco’s modified Eagle’s medium (DMEM) that was supplemented with 100 U/ml aqueous penicillin, 100 mg/ml streptomycin and 10% fetal bovine serum (FBS). Cells were maintained at 37 °C in a humidified atmosphere containing 5% CO2 and harvested with TrypLE (1X). Cell dissociation was deactivated with complete media. Cells were pelleted by centrifugation at 180 × g (relative centrifugal force, RCF) for 5 minutes and then re-suspended in complete media. The cell suspensions were used only when their viability as assessed by trypan blue exclusion exceeded 95% before use. Cell viability was determined and calculated as the number of viable cells divided by the total number of cells within the grids on a hemocytometer. The cell density of cell suspensions was determined using a hemocytometer. 0.1 mL of 0.4% of trypan blue solution was added to 0.1 mL of cells. The trypan blue solution and cell mixture was loaded into a hemocytometer and examined immediately under a microscope at low magnification. The number of blue staining cells and the number of total cells were counted. Cell viability should be at least 95% for healthy log-phase cultures.

### Quantitative real-time polymerase chain reaction (qRT-PCR)

Total RNA extraction was performed using the ARCTURUS PicoPure RNA Isolation Kit (Applied Biosystems, USA). The kit was designed to recover high-quality total RNA consistently from fewer than ten cells and even from a single cell. Total RNA extraction was executed as directed by the manufacturer. Total RNA was measured using Qubit RNA reagents (Invitrogen, USA). Measurements were conducted using the Qubit 3.0 Fluorometer (Invitrogen, USA). All the RNA samples did not undergo more than two freeze-thaw cycles to avoid any potential nucleic acid degradation. Total RNA reverse transcription (RT) was performed using the High-Capacity cDNA Reverse Transcription Kit (Applied Biosystems, USA). After reverse transcription, the cDNAs were diluted by 10-fold before amplification. cDNA amplification was performed using KiCqStart SYBR Green qPCR ReadyMix (Merck, Germany). The mRNA primer designs (Sigma-Aldrich, USA) are listed in the Supplementary Table S9a. All real-time PCR reactions were performed in triplicates using a SmartCycler automated real-time PCR system (Cepheid Inc., USA). Supplementary Table S9b sets the thermal cycling conditions for amplification. All target mRNA expressions were normalized to reference gene GAPDH. Relative mRNA levels were calculated using the –ΔΔCt method.

### Immunofluorescence

10 μL of cell suspensions that were retrieved from the microfluidic chip device as aforementioned were fixed with equal volume of 4% paraformaldehyde (PFA) and then dispensed on a poly-L-lysine coated glass slide (Sigma-Aldrich, USA). The cells were incubated at room temperature for 15 minutes. The fixed cells were washed with 1X PBS and then permeabilized with 0.1% Triton-X 100 at room temperature for 10 minutes. The cells were washed with 1X PBS and then blocked with 4% Bovine Serum Albumin (BSA) for 1 hour at room temperature. The cells were washed with 1X PBS and then incubated with primary antibody cocktail in 2% BSA at room temperature for 2 hours (Supplementary Table 10). The cells were washed twice with 1X PBS and then incubated with secondary antibody cocktail in 2% BSA at room temperature for 1 hour (Supplementary Table 10). The cells were washed twice with 1X PBS and then counterstained with ProLong Gold Antifade Mountant with DAPI (Invitrogen, USA). The slide was mounted with a cover slip and the cells were visualized using the Leica TCS SP8 confocal laser scanning microscopy platform (Leica Microsystems, Germany).

### Transit time analysis

Images acquired using the Basler acA800–510um USB 3.0 camera (Basler AG, Germany) of cells crossing the micro channels of constrictions at 100 frames per second from microfluidic experiments were analyzed with ImageJ Version 1.51. The Mtrack2 plugin was installed on ImageJ. It was used to track the positions of a cell cross a channel with constrictions (Fig. 2a, see also Supplementary Videos S11, S12). First, the entire image sequences were converted to mask. Then, the outline of the channel was subtracted from each image sequence. Finally, the positions of the cell were measured from each image sequence. This method automatically churned the measurements of the position vector of the cell that allowed the calculations of cell velocity and cell residence time. This method is only effective when there is only one cell crossing a channel from beginning to end. Thus, manual tracking was done on image sequences that had more than one cell crossing the channel within a single time frame.

### Cell morphological analysis

All acquired images were analyzed with ImageJ Version 1.51. Regions of interest (ROIs) were used to define specific parts (cell and nuclear boundaries) of an image that was processed independently. Only the pixels within any defined ROI were included in the calculations when measured. ImageJ’s set measurements function allowed the generation of the following data: area, circularity, roundness, aspect ratio and integrated density. This method was also used to measure the protein presence and its localization in a cell. Integrated Density is the product of “Area” and “Mean Gray Value”.

### Statistical analysis

All statistical hypothesis testing was conducted using the GraphPad Prism 8 software (GraphPad Software Inc., USA). An alpha of 0.05 was used as the cut-off for significance. The Kolmogorov-Smirnov test was used to compare two independent groups. This method was used to compare all data on transit time, nucleus morphology and γ-H2AX protein. The two-way analysis of variance (ANOVA) followed by Holm-Sidak multiple comparisons post hoc test was used to compare relative expression of every transcript between two groups (uncirculated and circulated) of two cell types (SK-BR-3 and MDA-MB-231). This method was also used to compare relative expression of nine transcripts between two groups (unconfined and every confined) for SK-BR-3 and MDA-MB-231.

## Supplementary information

Supplementary Information.

Supplementary Video S11.

Supplementary Video S12a.

Supplementary Video S12b.

Supplementary Video S12c.

Supplementary Video S12d.

## Data Availability

The data that support the findings of this study are openly available in figshare at 10.6084/m9.figshare.11340416 and 10.6084/m9.figshare.11340401.
