# Supplementary material for: Fluid shear stress coupled with narrow constrictions induce cell type-dependent morphological and molecular changes in SK-BR-3 and MDA-MB-231 cells
Source: Sci Rep. 2020 Apr 14;10:6386. doi: 10.1038/s41598-020-63316-w (PMC7156718; doi:10.1038/s41598-020-63316-w)
Supplement: Supplementary file 1 — Supplementary Information. [file 41598_2020_63316_MOESM1_ESM.pdf]

Supplementary Figure S1

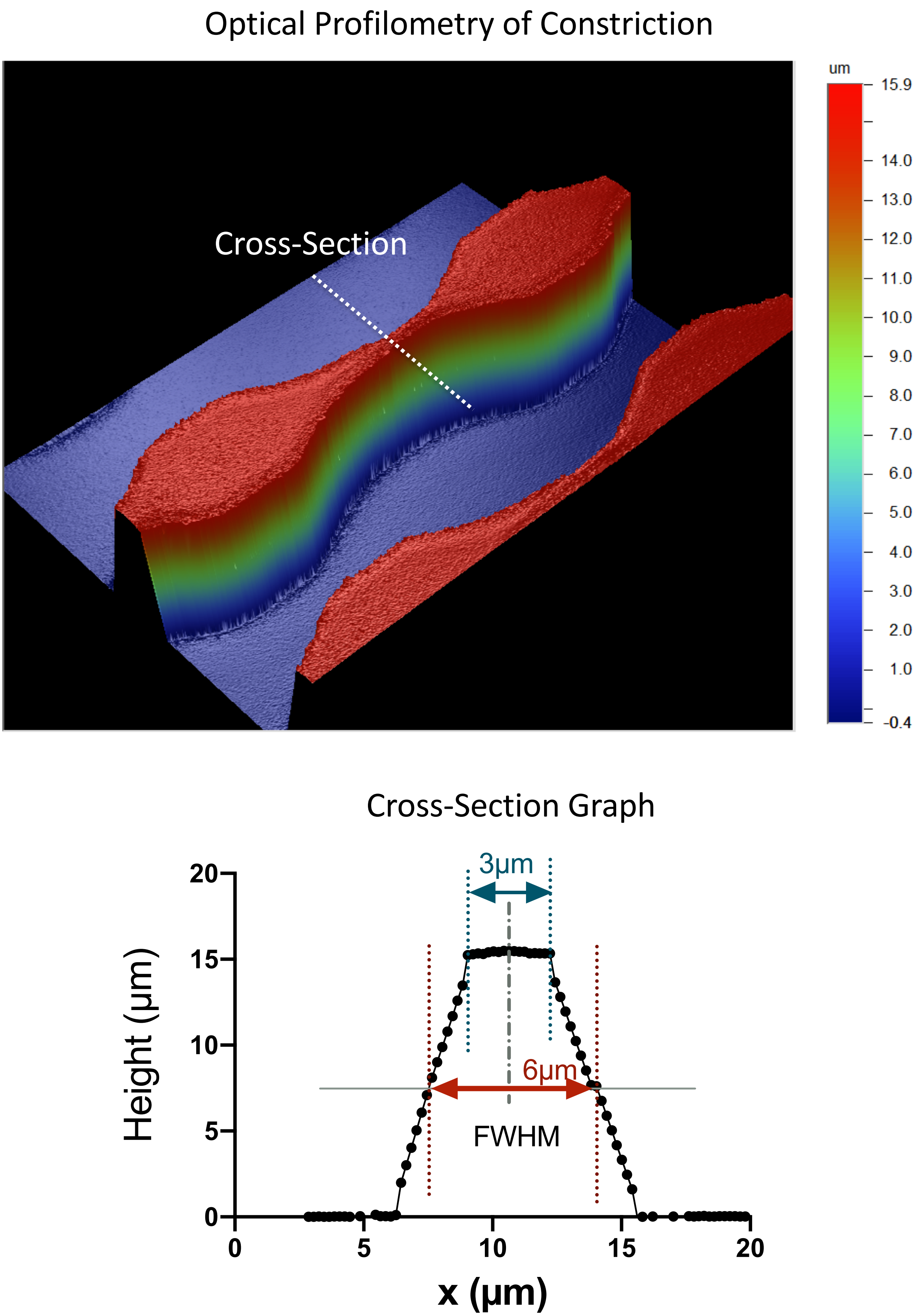

Supplementary Figure S2

COMSOL Simulation of Velocity of Various Microfluidic Devices at 10 kPa

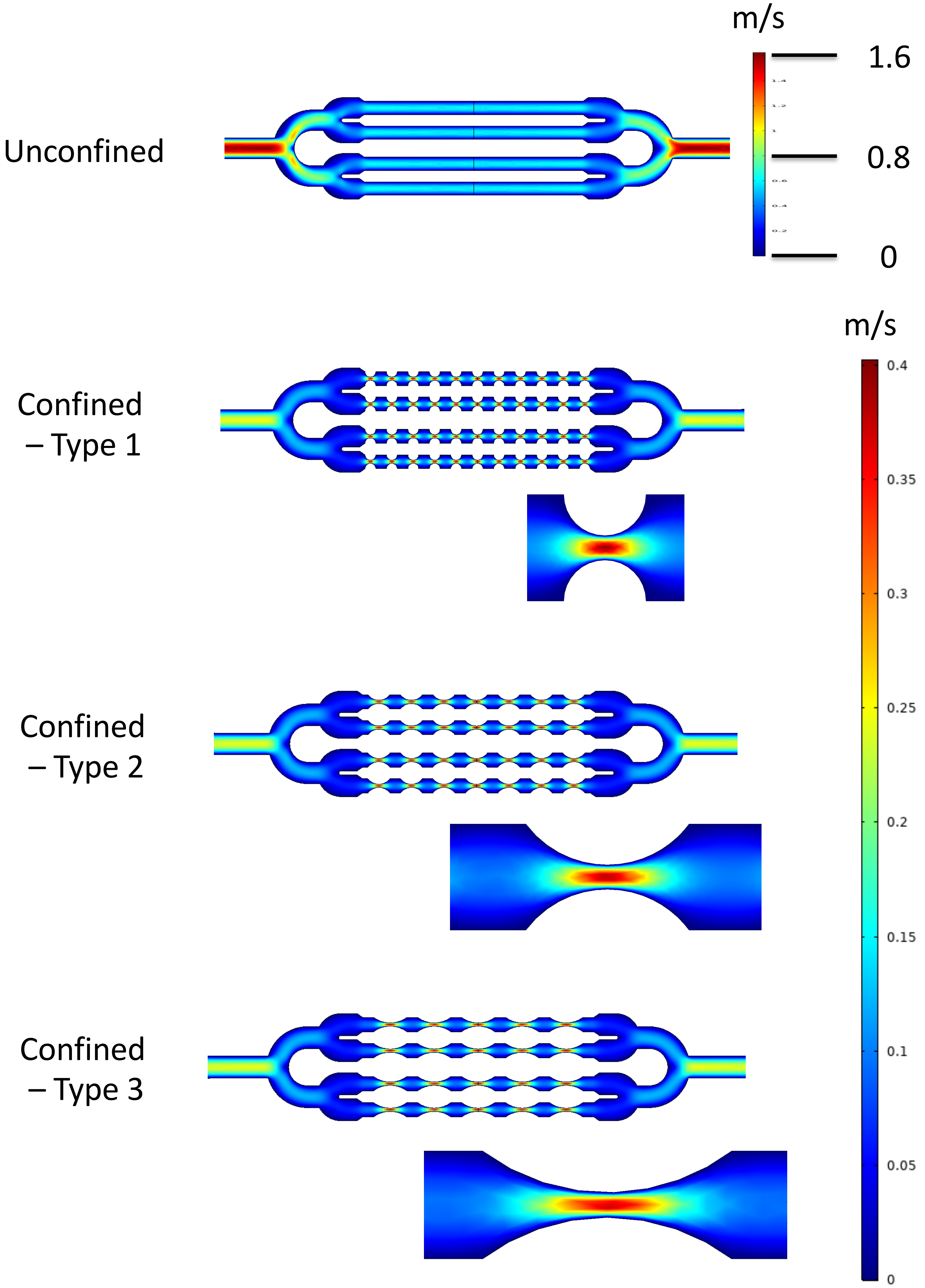

Supplementary Figure S3

Schematic of the Overall Experimental Set-up

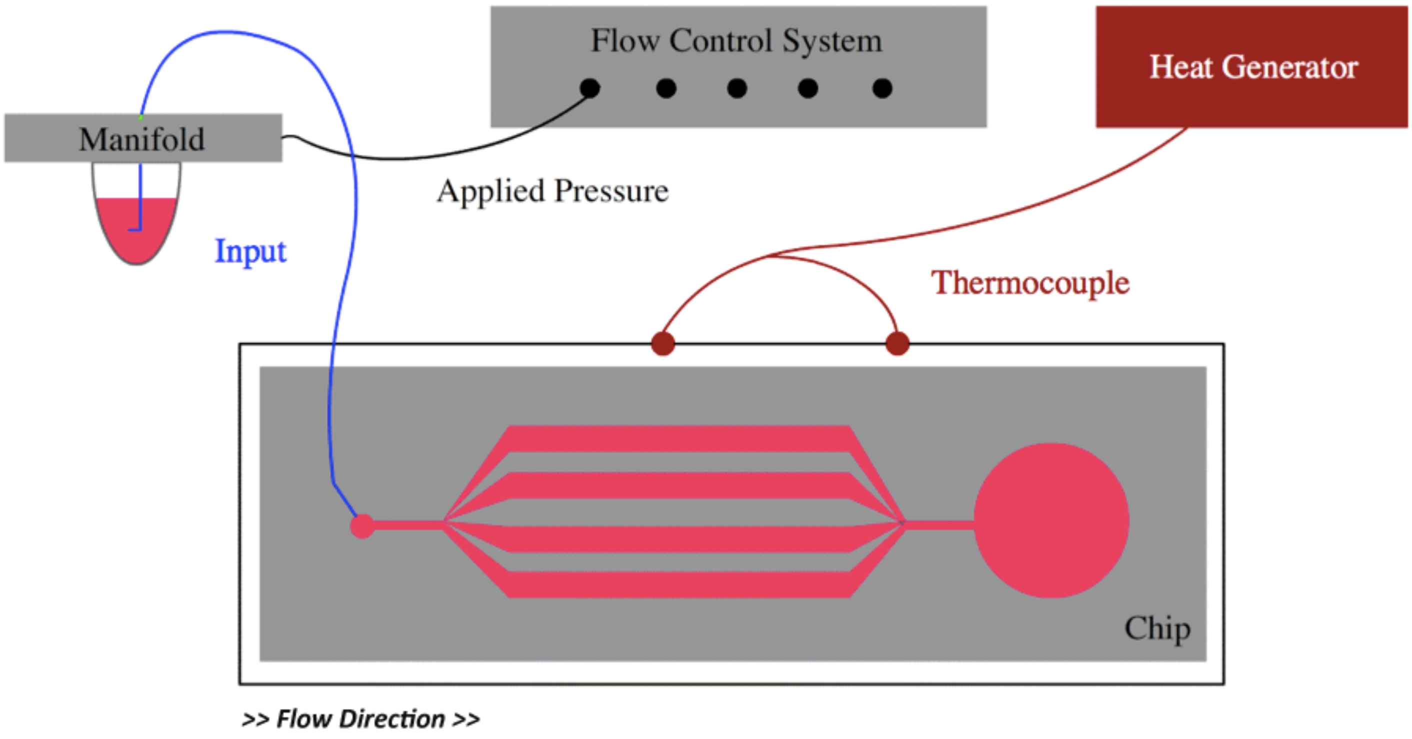

Chip is ready for cells and mounted on a 37°C stage.

Supplementary Figure S4

Representative Subset of Nuclei Images of SK-BR-3 Cells Post-Circulation

Control

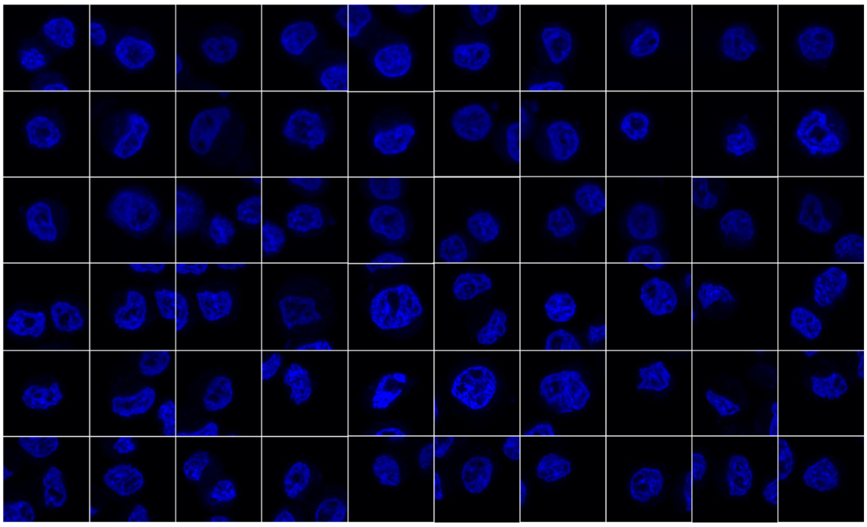

Unconfined

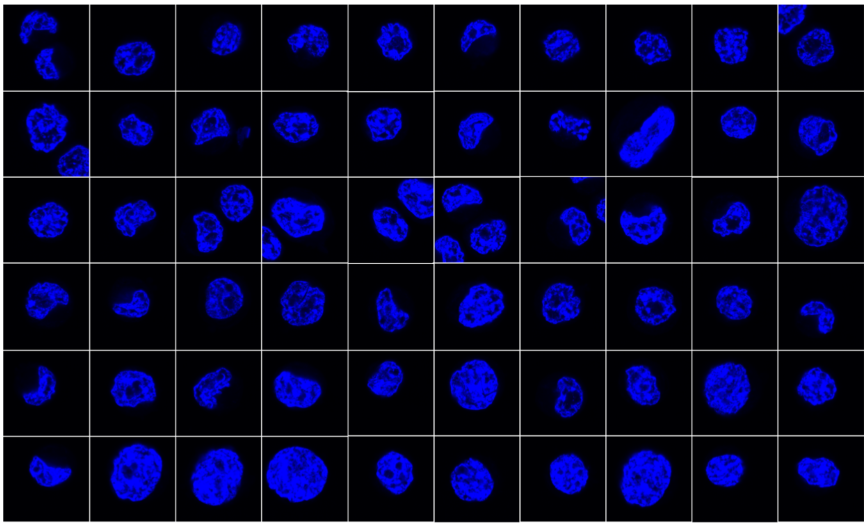

Confined

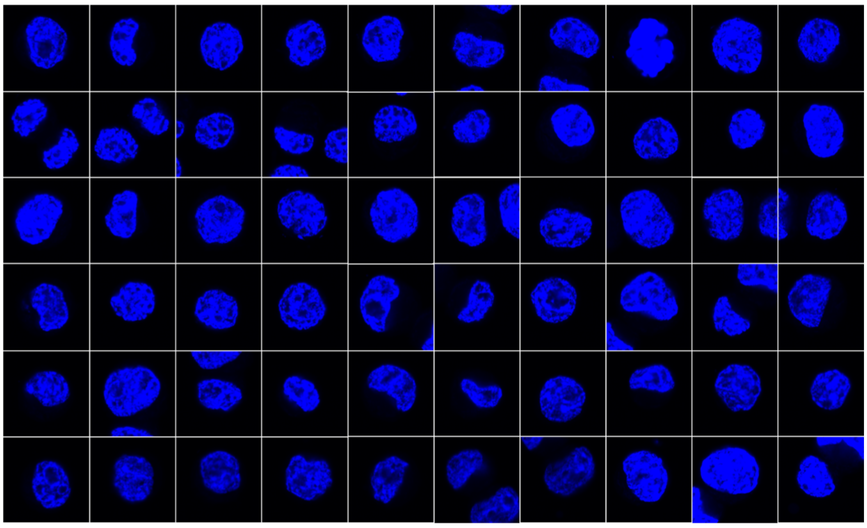

Supplementary Figure S4 (continued)

Representative Subset of Nuclei Images of SK-BR-3 Cells Post-Circulation

Confined – Type 1

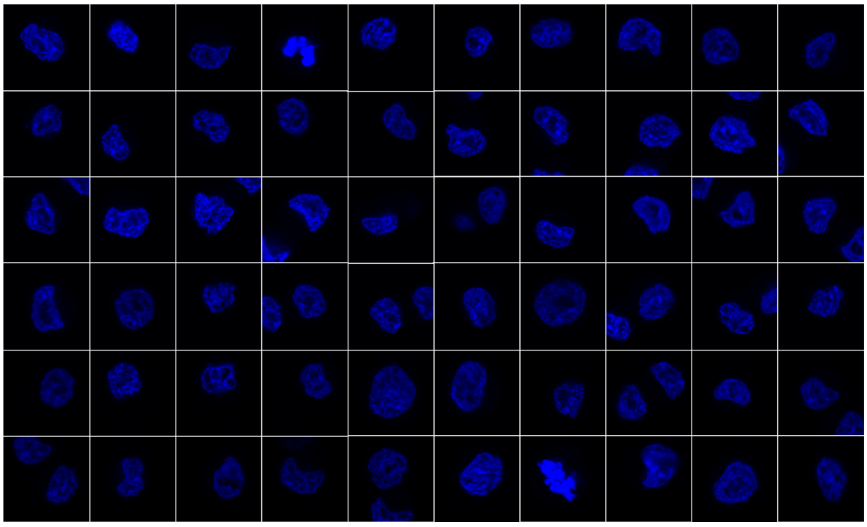

Confined – Type 2

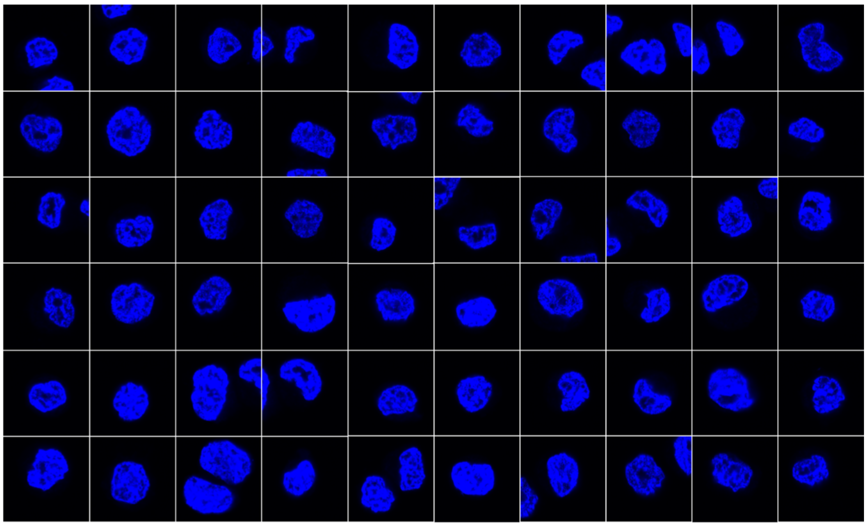

Confined – Type 3

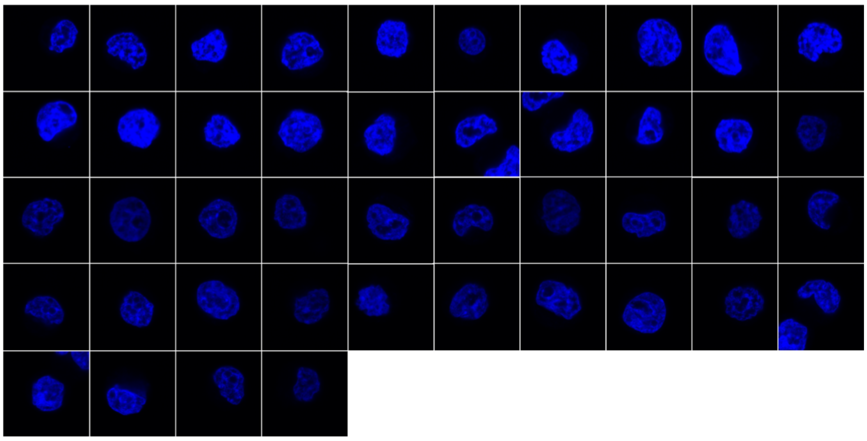

Supplementary Figure S5

Representative Subset of Nuclei Images of MDA-MB-231 Cells Post-Circulation

Control

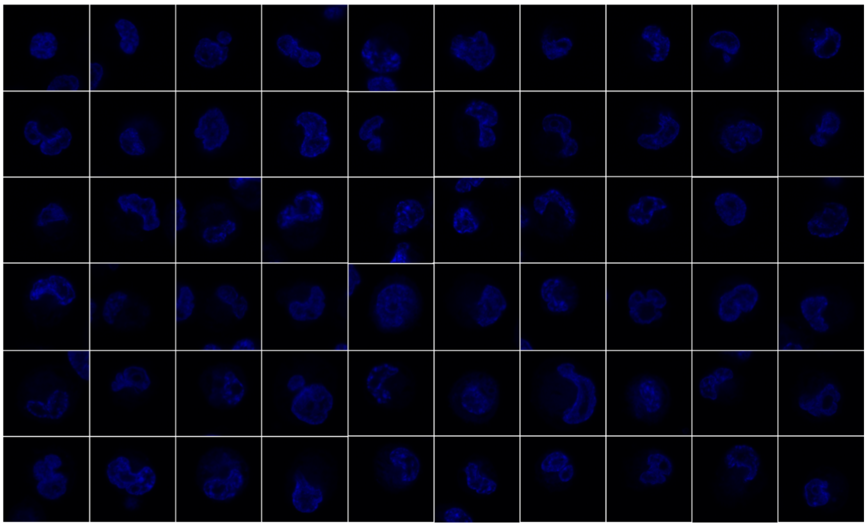

Unconfined

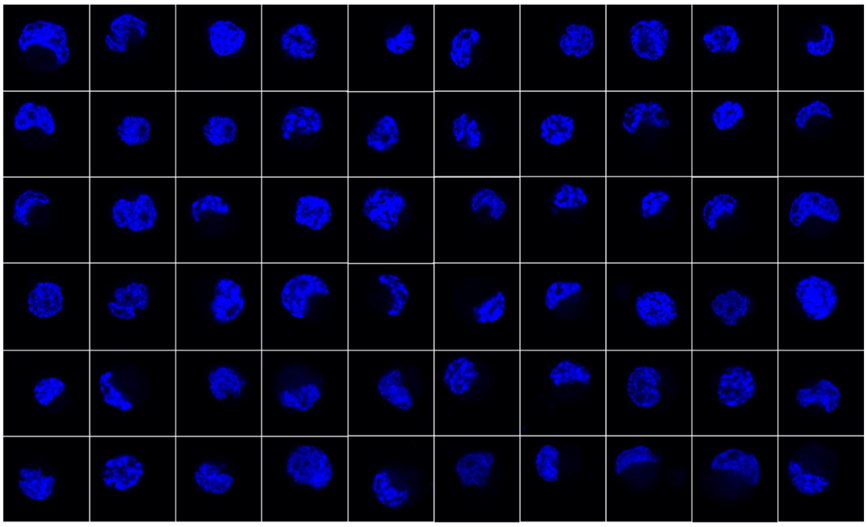

Confined

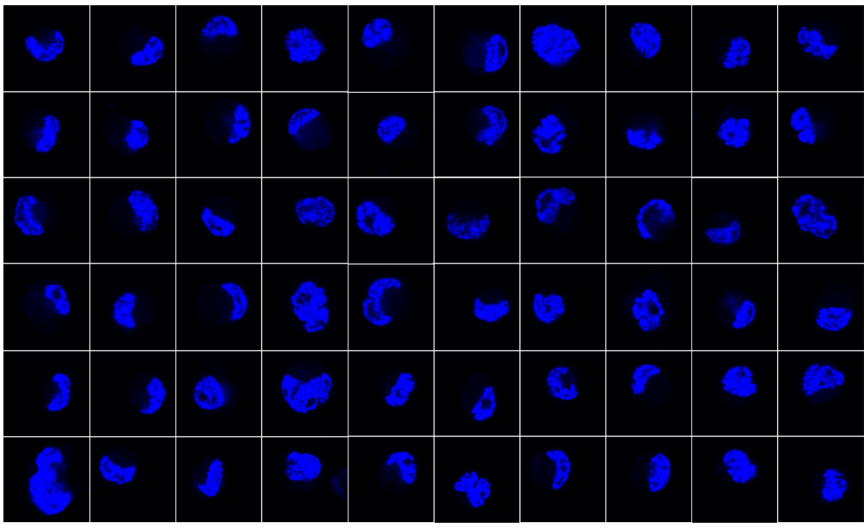

Supplementary Figure S5 (continued)

Representative Subset of Nuclei Images of MDA-MB-231 Cells Post-Circulation

Confined – Type 1

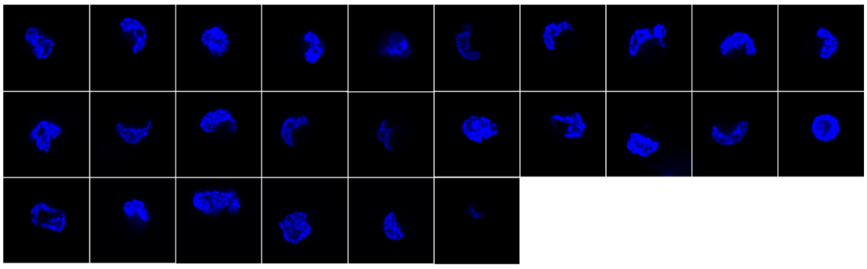

Confined – Type 2

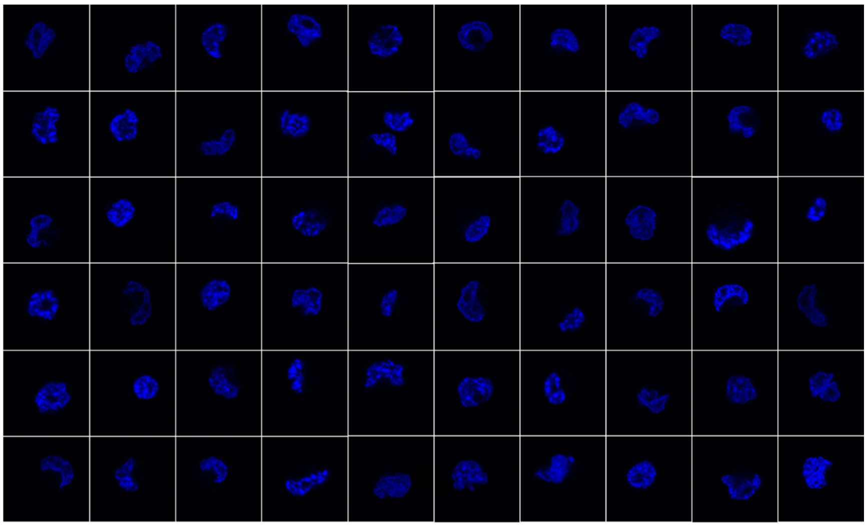

Confined – Type 3

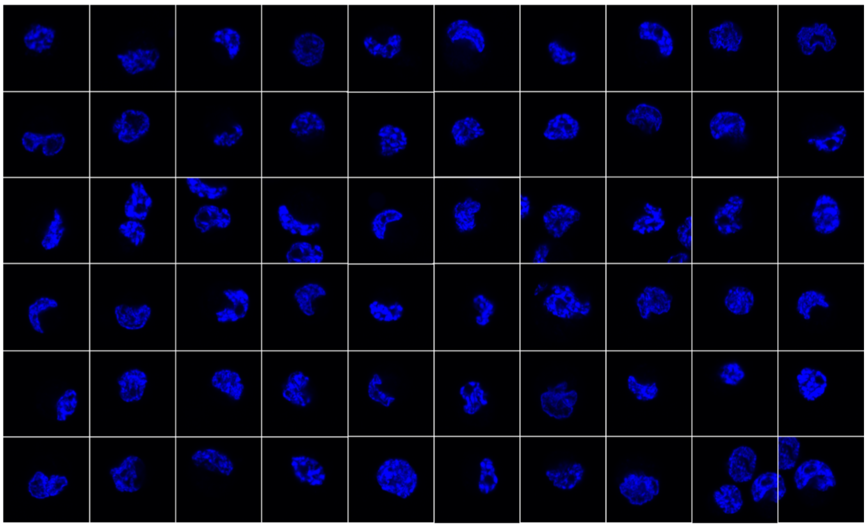

Supplementary Figure S6

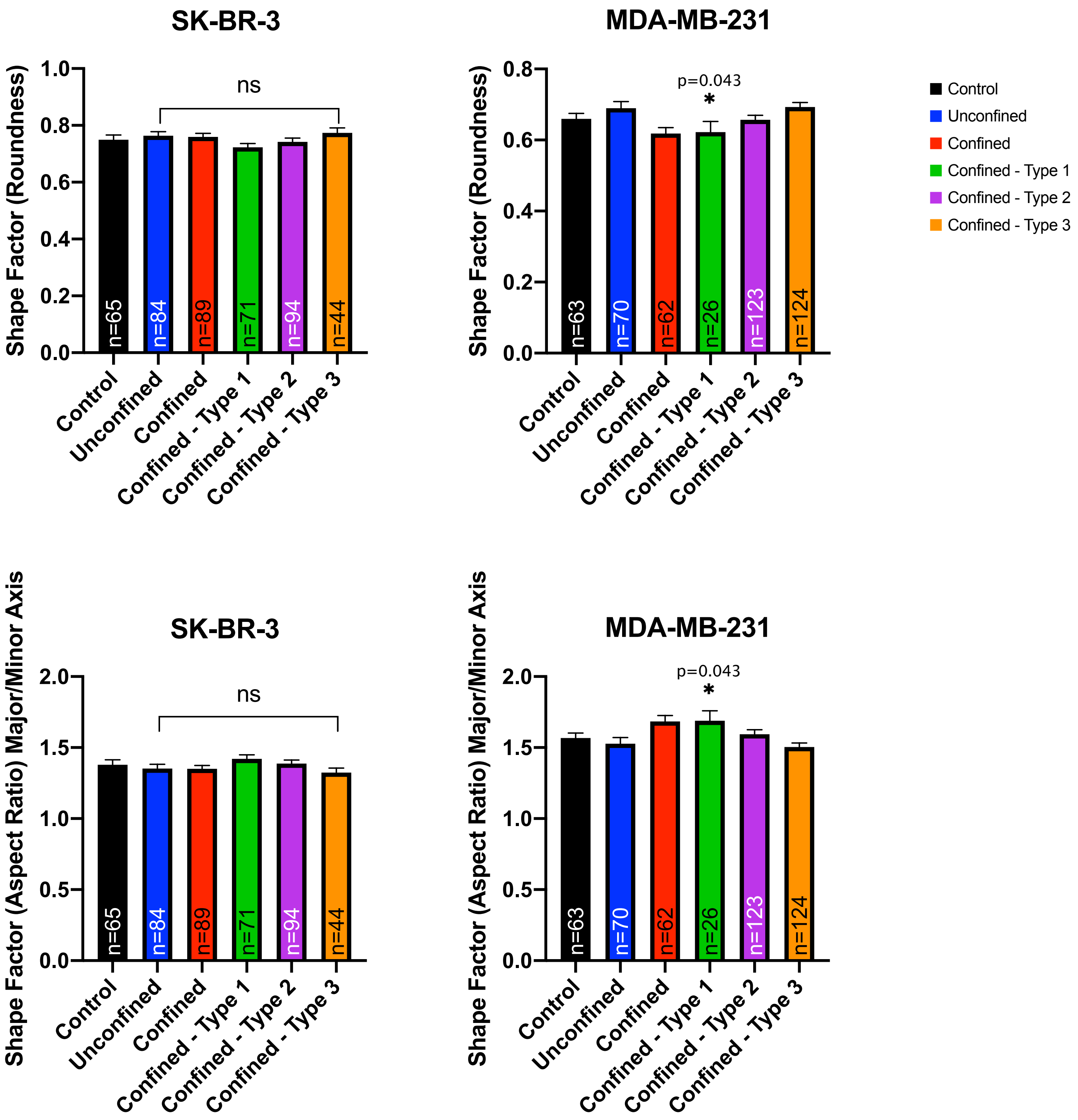

Supplementary Figure S6 Morphological analysis of SK-BR-3 and MDA-MB-231 cells post-circulation, -confinement and –constrictions at a constant applied pressure of 10 kPa. Quantifications of shape factor (roundness) and shape factor (aspect ratio) major/minor axis of SK-BR-3 and MDA-MB-231 nuclei. Error bars represent standard error of mean. Kolmogorov-Smirnov test. *, significance level at 0.033; **, significance level at 0.002; ***, significance level < 0.001; ns, not significant.

Supplementary Figure S7

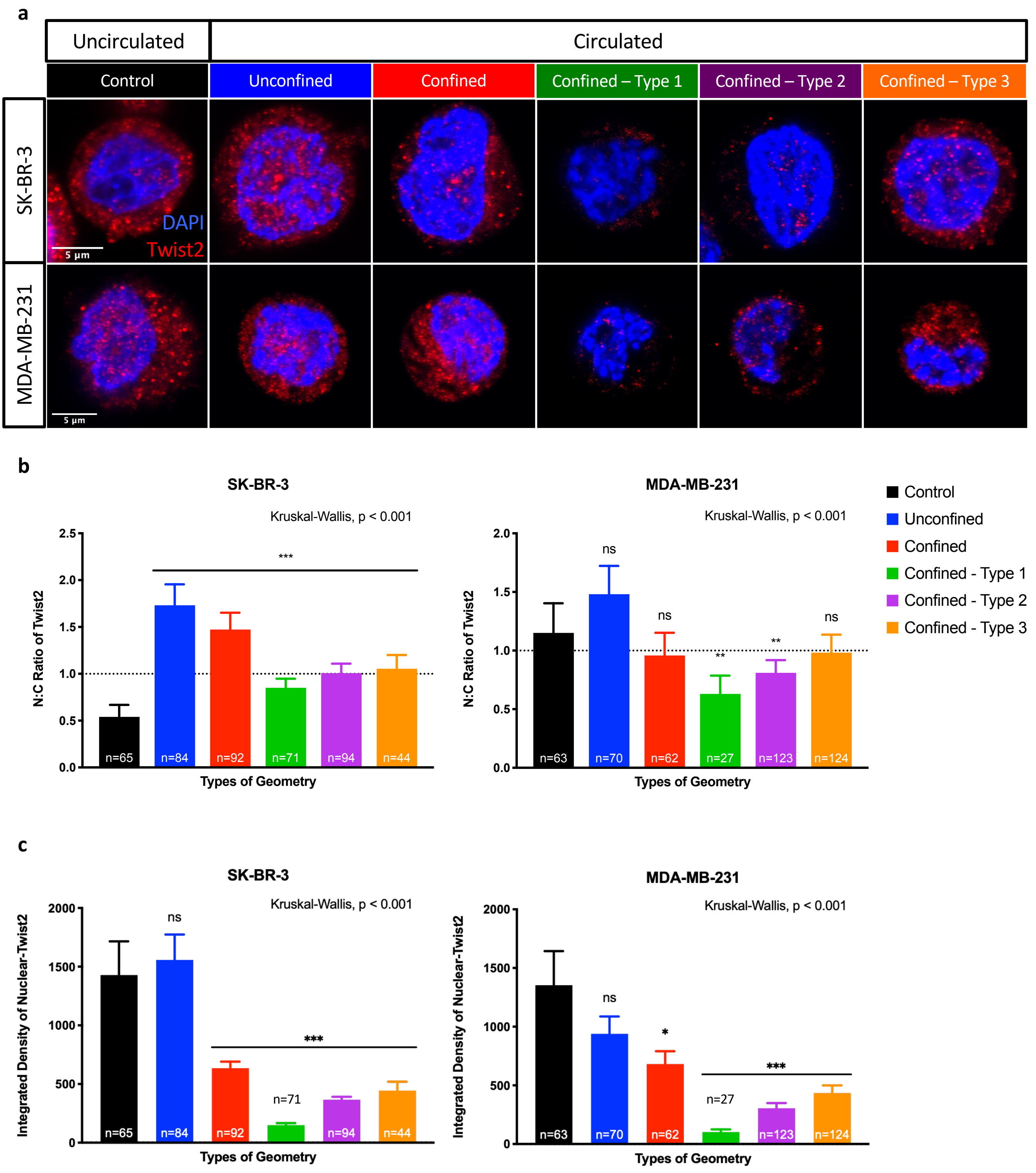

Supplementary Figure S7 Immunofluorescent analysis of SK-BR-3 and MDA-MB-231 cells post-circulation, -confinement and -constrictions at a constant applied pressure of 10 kPa using Twist2 antibody. **(a)** DAPI (blue) and Twist2 (red) staining of SK-BR-3 (top row) and MDA-MB-231 (bottom row) cells. **(b)** Quantifications of nuclear to cytoplasmic ratios of Twist2 integrated densities. Cytoplasmic co-localization – N:C ratio < 1; nuclear co-localization – N:C ratio > 1. **(c)** Quantifications of total Twist2 integrated densities. Error bars represent standard deviation. Dunn's multiple comparisons post hoc test. *, significance level at 0.033; **, significance level at 0.002; ***, significance level < 0.001; ns, not significant. Note: while the immunostaining Twist2 signals are significantly reduced in confined and constricted conditions, we find exactly the contrary for Twist2 gene expression. Similar discrepancies has been highlighted in a recent review of the literature, showing that there could be a delay between gene expression and protein expression. (Liu, Y., Beyer, A., & Aebersold, R. (2016). On the dependency of cellular protein levels on mRNA abundance. Cell, 165(3), 535-550.)

Supplementary Table S8

a

Thickness (µm)	Spin-coating Time (s)	Spin-coating Speed (rpm)	Soft Bake Time (min)
**20**	30	1000	3.5
**15**	30	1500	3

b

Thickness (µm)	Exposure Energy (mJ/cm²)	Exposure Time (s)	Post Exposure Bake Time (min)
**20**	145	16	4.5
**15**	140	17	4

c

Thickness (µm)	Development Time (min)	Shaker Speed (rpm)
**20**	3.5	100
**15**	3	100

Supplementary Table S8 Parameters for optical photolithography. **(a)** Conditions for spin-coating and soft bake. **(b)** Optimal exposure dosage and post exposure bake. **(c)** Development times for SU-8 developer.

Supplementary Table S9

a

Gene Symbol	Gene Name	Forward Sequence (5' – 3')	Reverse Sequence (5' – 3')
**VIM**	Vimentin	GGAAACTAATCTGGATTCACTC	CATCTCTAGTTTCAACCGTC
**CDH1**	E-cadherin	CCGAGAGCTACACGTTC	TCTTCAAAATTCACTCTGCC
**CDH2**	N-cadherin	ACATATGTGATGACCGTAAC	TTTTTCTCGATCAAGTCCAG
**SNAI1**	Snail1	CTCTAATCCAGAGTTTACCTTC	GACAGAGTCCCAGATGAG
**SNAI2**	Snail2 / Slug	CAGTGATTATTTCCCCGTATC	CCCCAAAGATGAGGAGTATC
**TWIST1**	Twist1	CTAGATGTCATTGTTTCCAGAG	CCCTGTTTCTTTGAATTTGG
**TWIST2**	Twist2	CATAGACTTCCTCTACCAGG	CATCATTCAGAATCTCCTCC
**ZEB1**	ZEB1	AAAGATGATGAATGCGAGTC	TCCATTTTCATCATGACCAC
**ZEB2**	ZEB2	AAGACTTCGCAGATCGAG	TGATAAGAGCGGATCAGATG
**GAPDH**	GAPDH	ACAGTTGCCATGTAGACC	TTGAGCACAGGGTACTTTA

b

	PCR Stage	Temperature	Time
**Stage 1**	Hold	95°C	10 min
**Stage 2**	Cycle (40 Cycles)		
	Denature	95°C	15 s
	Anneal/Extend	60°C	60 s
**Stage 3**	Melt Curve (Dissociation Stage)	95°C	10 s
		0.2°C Increments	
		60°C to 95°C	10 s

Supplementary Table S9 The mRNA primers and thermal cycling conditions for amplification. **(a)** The mRNA primers. **(b)** Thermal cycling conditions for cDNA amplification.

Supplementary Table S10

Primary Antibody Cocktail				
**Antibody**	Catalog	Initial Concentration	Host	Final Dilution
**TWIST2 Polyclonal Antibody**	PA5-66539, ThermoFisher Scientific	0.3 mg/mL	Rabbit	1:200
**Phospho-Histone H2A.X (Ser140) Monoclonal Antibody (3F2)**	MA1-2022, ThermoFisher Scientific	1 mg/mL	Mouse	1:500
**Secondary Antibody Cocktail**				
**Antibody**	Catalog			Final Dilution
**Goat anti-Rabbit IgG (H+L) Secondary Antibody, Alexa Fluor® 594 conjugate**	R37117, ThermoFisher Scientific			1:200
**F(ab')2-Goat anti-Mouse IgG (H+L) Secondary Antibody, Alexa Fluor® 488 conjugate**	A-11017, ThermoFisher Scientific			1:200

Supplementary Table S10 Primary and secondary antibody cocktails.

Supplementary Video S11

An SK-BR-3 cell transiting a Confined – Type 2 microfluidic device at a low applied pressure of 1 kPa acquired at 100 frames per second.

Supplementary Video S12

a) An MDA-MB-231 cell transiting a Confined microfluidic device at 10 kPa acquired at 100 frames per second.

b) An MDA-MB-231 cell transiting a Confined – Type 1 microfluidic device at 10 kPa acquired at 100 frames per second.

c) An MDA-MB-231 cell transiting a Confined – Type 2 microfluidic device at 10 kPa acquired at 100 frames per second.

d) An MDA-MB-231 cell transiting a Confined – Type 3 microfluidic device at 10 kPa acquired at 100 frames per second.
